# Wear Evolution of the Glass Fiber-Reinforced PTFE under Dry Sliding and Elevated Temperature

**DOI:** 10.3390/ma12071082

**Published:** 2019-04-02

**Authors:** Ruoxuan Huang, Siqi Ma, Meidi Zhang, Jie Yang, Dehong Wang, Liang Zhang, Jiujun Xu

**Affiliations:** 1 Department of materials science and engineering, Dalian Maritime University, Dalian 116026, China; masiqi1020@163.com (S.M.); hyb0509@gmail.com (M.Z.); 2 Key Lab of Ship-Maintenance & Manufacture, Dalian Maritime University, Dalian 116026, China; yangjie_jx@foxmail.com (J.Y.); wdhfree@163.com (D.W.); 3 Shanghai Micropowers Ltd., Shanghai 201203, China; zhangliang@micropowers.com

**Keywords:** PTFE, glass fiber reinforced, wear evolution, mechanisms, elevated temperature

## Abstract

The wear evolution of the glass fiber reinforced Polytetrafluoroethylene (PTFE) sliding against duplex steel at elevated temperature was investigated using the interrupted wear tests coupling with the worn surface observations. The morphological changes of the PTFE composite during the sliding were related to the variation of the tribological properties to analyze the underlying wear mechanisms. Results show that the coefficient of friction and wear rate change with the increase of temperature. During the sliding, three regions can be identified regardless of the temperature. The high temperature is beneficial to the formation of tribo-film. The sequence of wear evolution is PTFE removal, load transfer to glass fiber, and minor formation of tribo-film for the low temperature condition. For high temperatures, the wear behaviors are more complicated. The different phenomena include the third body abrasion, flake delamination of PTFE matrix, scratching and reformation of transfer film on the counterface, and the filling of the large scale PTFE groove. These behaviors may dominate the different stages in the stable region, but occur simultaneously and cause the dynamic steady wear. As a result, the wear rate at 200 °C is slightly fluctuant.

## 1. Introduction

Polytetrafluoroethylene (PTFE) possesses advantageous properties, such as a low coefficient of friction, self-lubrication, and chemical inert, as well as relatively good thermal stability, etc. [1]. A combination of these properties results in the use of the PTFE in the tribological applications. However, the mass loss of PTFE caused by the poor anti-wear property and abrasive resistance limits its broad utility. In order to overcome the problem, attention has been attracted to the embedding fillers to make PTFE into composites. Up to now, various nano- and micro-meter inorganic fibers and metallic oxides, as well as other particles such as MoS_2_, were used at the reinforcement phase in the PTFE to enhance the friction and wear performance, and an apparent improvement has been obtained. Among the fillers, glass fiber, owing to its low cost and light weight, as well as its good enhancement, is considered as the prevalent candidate blended in PTFE. It is commonly used in different percentages and is sometimes combined with other fillers [2]. T. A. Blanchet found that glass fibers reduce wear by interrupting subsurface deformation and crack propagation, which would otherwise lead to large wear sheets under severe sliding conditions [3]. S. S. Kim and M. W. Shin reported that short glass fibers with normal orientation to the sliding interface were conducive to the tribological properties of the PTFE composite [4]. M. Conte suggested that the use of glass fibers in a PTFE matrix would improve the tribological properties by reducing the abrasiveness [5]. On the other hand, the interfacial adhesion between the fibers and PTFE was reported to be another vital factor that affects the performance of PTFE composites [6], and researchers have found that silane and rare earth are the effective coupling agents to improve the tribological performance of the PTFE-based composites [6,7]. Most of the studies were performed at the room temperature with the changes of sliding velocity and loading. However, many applications, such as compressor piston rings and bearings, require an understanding of the tribological properties at elevated temperatures [8]. To our best knowledge, however, few studies have focused on this area.

The stable friction force and the wear loss after a period of sliding were widely used to evaluate the tribological performance of the PTFE-based composites. However, to further optimize the tribological properties of the materials, it is necessary to fully understand the wear evolution and the associated mechanisms. Usually, the worn surface of the composite and the counterface material after wear testing can be examined to explore the microstructural and chemical changes in the final stage and to analyze the possible wear behaviors [9,10,11]. However, the post-test observations neglect the effects of elastic recovery, shrinkage, and crack closure in a PTFE matrix, and thus cannot reveal the real-time wear process. Therefore, the wear evolution needs to be tracked to reveal the wear process of the PTFE-based composites.

The aim of this work is to investigate the tribological properties and wear evolution of the glass fiber-reinforced Polytetrafluoroethylene (GFRPTFE) sliding against duplex steel. The periodical interrupted tests performed on an in-house developed tribo-tester combined with the laser scanning microscopy observations were conducted to directly trace the surface morphological changes of the GFRPTFE and steel specimens. The wear evolution during the sliding was linked to the variation of the tribological properties to further analyze the underlying wear mechanisms.

## 2. Materials and Methods

### 2.1. Materials and Specimens

The GFRPTFE specimens were purchased from Saint-Gobain Crossflon Inc and marketed under the trademark of RULON^®^ AR. The materials were purposely designed and manufactured for seals and applications requiring high properties. The detailed engineering information and physical data provided by the supplier are listed in Table 1.

**Figure 1:**
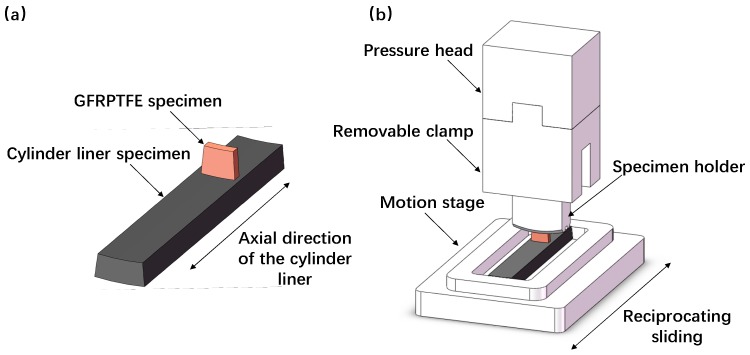
The schematic of the interrupted wear test configurations. (**a**) The friction pairs and (**b**) the arrangement of the test rig.

Differential scanning calorimetric (DSC, Netzsch, Selb, Germany) test of the GFRPTFE specimen was conducted at a heating rate of 10 °C/min in liquid nitrogen atmosphere in the temperature range from 0 °C to 300 °C with a thermal analyzer (the applicable temperature of the PTFE ranges from −240 °C to 288 °C; this study focused on the elevated temperature, so the temperature interval of 0 °C to 300 °C was selected, and this interval fully covered the wear test temperatures). Figure 1a shows the schematic of the test specimens. The upper specimens were directly machined from the commercial GFRPTFE. The hardness of the GFRPTFE specimen is 62 HD, implying that the material is soft and has low indentation resistance. As one typical application of the GFRPTFE is the sealing ring in the reciprocating engine [12], the duplex stainless steel obtained from the cylinder liner of an actual stirling engine was selected as the lower counterface material. Prior to the wear tests, the inner surface of the cylinder liner was treated by the nitriding and fine grinding, resulting in a high hardness of 1102 HV_0.1_ and surface roughness of Ra = 0.14 μm. The GFRPTFE and counterface specimens were machined into the required dimensions listed in Table 2. Note that the inner diameter of the cylinder liner specimen is exactly equal to the outer diameter of the GFRPTFE specimen, thus perfect mating between the two specimens can be achieved during the sliding.

### 2.2. Friction Testing

The wear tests were performed on an in-house-designed contraposition-reciprocating tribology device as illustrated in Figure 1b. The test arrangement is nominally identical to those of the reciprocating tribo-tester detailed in the previous reports [13,14,15]. The GFRPTFE specimen placed between the cylinder liner specimen and the pressure head was mounted on the holder, which was connected with a horizontally-fixed force sensor. During the sliding, the GFRPTFE specimen and the pressure head were kept still, whilst the heated stage moved to and fro. The applied normal force was 28.8 N, resulting in the contact pressure of 1.6 MPa. The sliding speed and stroke length were 300 rpm and 50 mm. The variable experiment parameter is the temperature; the tests were conducted at 50 °C, 80 °C, 100 °C, 120 °C, 140 °C, 160 °C, 180 °C, and 200 °C without lubrication. After 15km sliding, the GFRPTFE specimens were taken out and cleaned for the measurement of weight loss, and the wear rate was calculated by dividing the weight loss by the sliding distance. Four repeat tests were carried out for each temperature.

### 2.3. Interrupted Wear Testing

A removable clamp connecting the pressure head and the specimen holder was used to enable the interrupted wear tests, which can track the wear evolution and the intermittent weight loss of the GFRPTFE specimen during the sliding, the wear tests were interrupted periodically (every 0.5 km sliding). Prior to the interrupted test, the preconditioned sliding without loading was performed between the GFRPTFE and liner specimens to achieve the uniform distribution of the pressure. After each interruption, the weight of the GFRPTFE specimen together with the removable fixture was measured by the balance such that the wear rate could be calculated and the specimen could be perfectly remounted for the sequential sliding. Moreover, the morphologies of the wear surface of the friction pair were observed by confocal laser scanning microscopy (CLSM, Olympus LEXT OLS4000, Olympus, Tokyo, Japan), and the chemical components of the cylinder liner specimen were examined by energy dispersive X-ray spectroscopy (EDS, Oxford Instruments, Oxford, United Kingdom) to investigate the transfer film caused by the friction. This study focused on the tribological performance of the GFRPTFE at elevated temperatures, thus 100 °C and 200 °C were selected as the experimental temperatures. The total sliding distance was 15 km.

## 3. Results

### 3.1. The Internal Microstructure of the GFRPTFE Specimen

**Figure 2:**
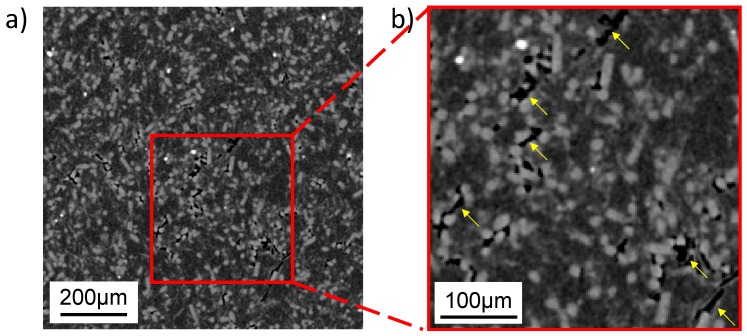
The representative X-ray micro-tomographic slice of the GFRPTFE specimen (**a**) Low magnification image and (**b**) high magnification image.

Figure 2 illustrates the 2D X-ray micro-tomography (XMT) slice of the GFRPTFE specimen. The XMT scan was performed by Versa XRM-500 (Zeiss, Oberkochen, Germany) at 35 kV and 110 mA, and used to examine the internal microstructure of the GFRPTFE specimen with a resolution of ~2.2 μm (Figure 2b). The glass fibers were randomly distributed in the PTFE matrix, resulting in a homogeneous microstructure of the composite. There were some white particles with a diameter of ~10 μm embedded in the matrix as well. From the prior knowledge, they are the as-fabricated Fe_2_O_3_ particles, which can be sintered on the steel surface and may contribute to the formation of the tribo-film [16,17]. As the densities of glass fiber (~2.5 kg/m^3^) and PTFE (~2 kg/m^3^) are much lower than Fe_2_O_3_ (~5.3 kg/m^3^), the Fe oxidation particles show the highest grey level in the XMT slice. In addition, the defects, as marked in yellow, can be clearly observed. Since the grey level of the defect is very close to zero, they are most likely the air voids.

### 3.2. Effect of Temperature on the Friction and Wear Rate of GFRPTFE

**Figure 3:**
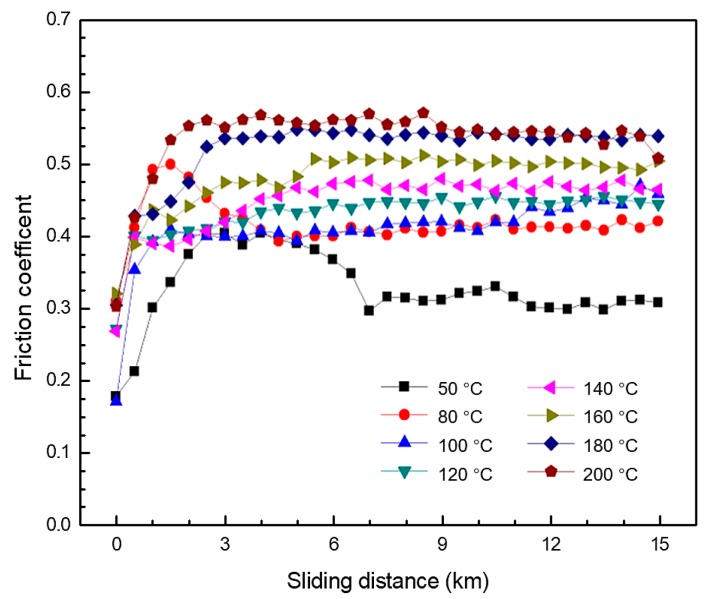
The representative friction coefficient versus sliding distance curves at different temperatures.

Figure 3 shows the representative friction coefficient curves of the GFRPTFE with various temperatures ranging from 50 °C to 200 °C for 15 km sliding. It can be found that the coefficient of friction increases with the temperature, and three regions can be identified in each curve.

I. Running-in region: An initial portion, with an approximately linear increase, corresponds to the wear of the top layer of pure PTFE (refer to Section 3.3. The sharp increase region is limited up to the sliding distance of 0.5 km for the elevated temperature (≥100 °C) and 1 km for 50 °C and 80 °C. 

II. Transition region: The non-linear increase of the friction coefficient arises after the linear portion, implying the change of friction condition caused by the load transfer from the PTFE matrix to the glass fiber. 

III. Stable wear region: This region is characterized by a near plateau of the friction coefficient in the curves after the load transfer is finished. The friction coefficient is stable, although it varies a lot with the temperature and can slightly increase, decrease, or maintain constant with the increase of sliding distance. The phenomenon is mainly attributed to the simultaneous wear behaviors: The load bearing of glass fiber, PTFE matrix softening, and ploughing, as well as the transfer film (refer to Section 3.3 and Section 3.4). The stable region determines tribological performance of the GFRPTFE, and plays the most important role in actual applications.

**Figure 4:**
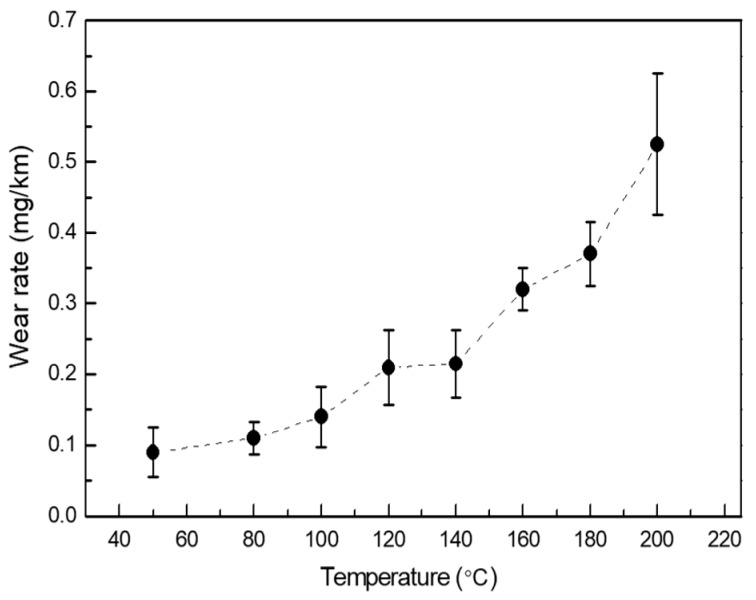
The measured wear rate of GFRPTFE after 15 km sliding at different temperatures.

The measured wear rate of GFRPTFE after 15 km sliding at different temperatures is shown in Figure 4. The wear rate of GFRPTFE increases with the temperature as well, similar to the fiber-reinforced PEEK composites reported by Mu [8]. The difference of the wear rate between 50 °C and 200 °C is about five times, which is much higher than that of the friction coefficient (less than two times). Note that the deviation of the measured wear rate at 200 °C is remarkably larger than other temperatures due to the complicated wear behaviors which will be discussed in the surface morphology observation of the worn specimen.

**Figure 5:**
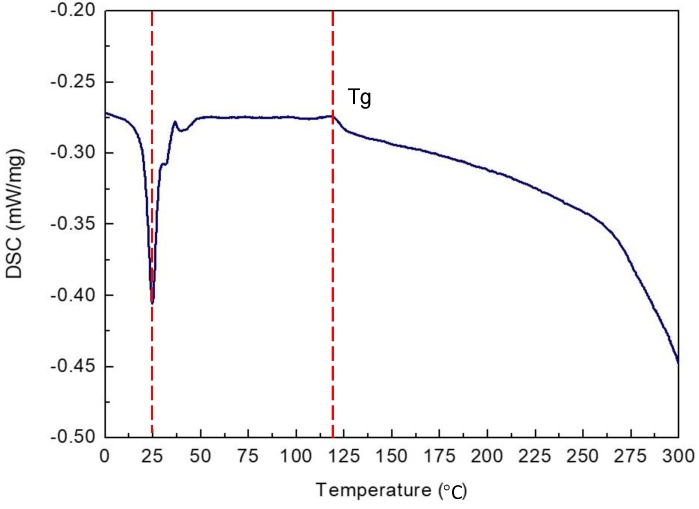
The differential scanning calorimetric (DSC) thermogram of the GFRPTFE.

The DSC thermogram (refer to Figure 5) indicates three characteristics of the GFRPTFE: (1) The peak at the temperature of ~25 °C is generally considered as the crystalline transition from the triclinic to the hexagonal phase [18]; (2) the higher temperature of ~121 °C causes the mobility of the rigid amorphous of PTFE [18], which directly results in a considerable increase of the wear rate measured at 120 °C. This is because the segmental motion of the polymer chain is activated after the glass transition temperature Tg of PTFE rigid amorphous fraction, and then the amorphous fraction presents the elastomer state and, accordingly, the mechanical properties of the PTFE matrix begin to degrade; (3) another universal agreement on PTFE is the melting temperature of ~320 °C. As the DSC measurement stops at 300 °C [19], only a part of the large endothermic peak corresponding to the Tm is shown in Figure 5. While the highest temperature for the tribology tests was 200 °C, much heat on the surface of the friction pair may be accumulated due to the high friction force. Thus the localized temperature on the GFRPTFE surface may be close to the Tm, and consequently severe localized softening of the PTFE matrix may occur.

**Figure 6:**
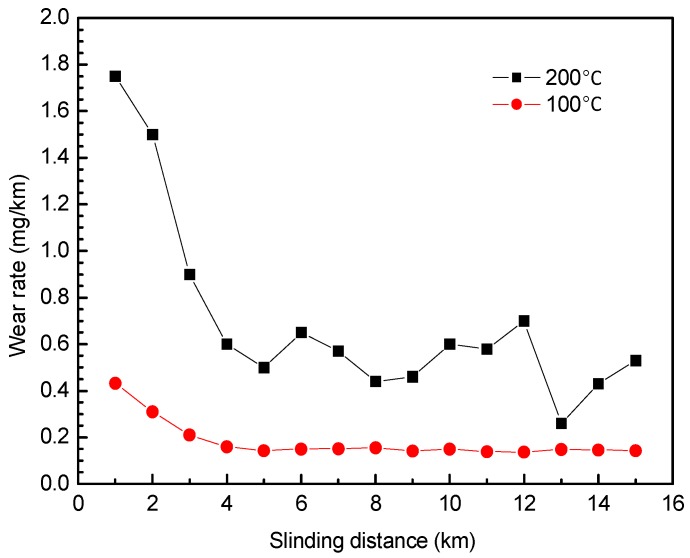
The wear rate of GFRPTFE versus sliding distance of the friction pair at 100 °C and 200 °C.

Figure 6 illustrates the variation of the wear rate with the sliding distance at selected elevated temperatures. Since the wear rate of GFRPTFE is too small to measure when the temperature is lower than 100 °C, only the results at 100 °C and 200 °C are presented. With the increase of sliding distance, the wear rate, which decreases until 5 km and then goes to a stable level, shows the inverse trend compared with the friction coefficient, regardless of the temperature. In the initial running-in and transition regions, the wear rate of GFRPTFE at 100 °C is much lower than that at 200 °C, and an even lower wear rate was obtained at 50 °C and 80 °C. Therefore, the low wear rate is most likely the reason that more sliding distance is needed to achieve the stable wear at low temperature conditions. In the stable region, the wear rate almost keeps constant for 100 °C. In contrast, the noticeable and irregularly fluctuant wear rate can be observed when the temperature is 200 °C, and this result agrees well with the great deviation of the four measured wear rates after 15 km sliding.

### 3.3. The Surface Wear Evolution of the GFRPTFE at Elevated Temperatures

**Figure 7:**
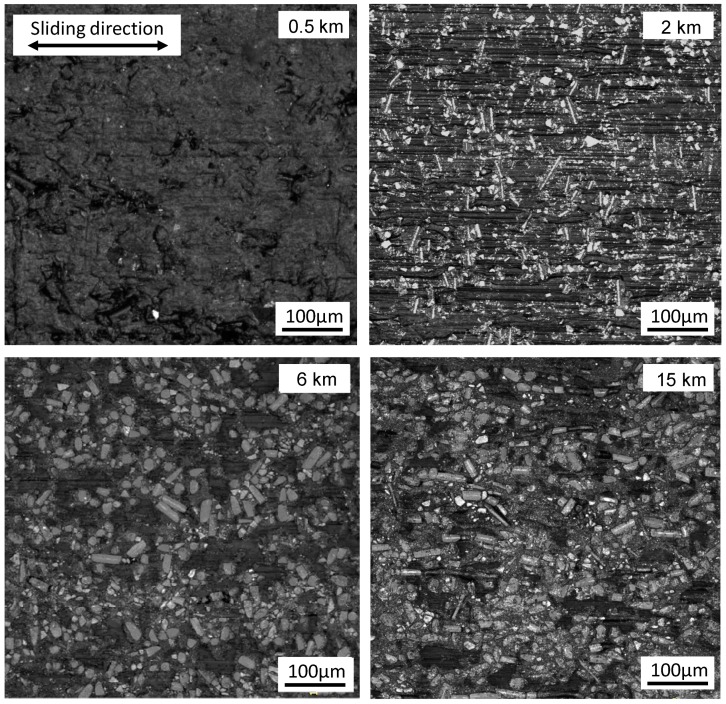
The wear evolution of the GFRPTFE sliding against duplex steel at 100 °C.

In order to trace the wear evolution of GFRPTFE, a few typical CLSM images containing the representative features were selected from the various sliding distances on the basis of the three identified regions. Figure 7 shows the wear evolution of the GFRPTFE specimen surface at 100 °C. The original appearance of the GFRPTFE specimen can still be observed in the image of 0.5 km, although the micro wear scars caused by the initial sliding have occurred on the surface. As no visible glass fiber can be found, the reinforced phase is still buried beneath the top layer of the PTFE matrix. With the sliding distance increased to 2 km where the wear rate has already got into the transition region, the micro scars become the macro furrows, which occupy the whole surface. Meanwhile, the glass fibers are gradually exposed after the removal of the covering PTFE is rubbed (see the image of 2 km in Figure 7), and then involved in the wear process. At this stage, most glass fibers are located individually in the matrix, and PTFE is still the majority component on the surface. Subsequently, the stable wear is achieved, and the glass fiber dominating surface is presented as illustrated in the image of 6 km. A great number of glass fibers become irregular in shape due to the wear of their exposed part, and orient randomly. At the late stage of the wear test (15 km), the CLSM image shows no essential difference, only the area ratio of the fiber is slightly larger than that of the image acquired at the beginning of the stable region, and the produced wear dusts are collected surrounding the glass fibers.

**Figure 8:**
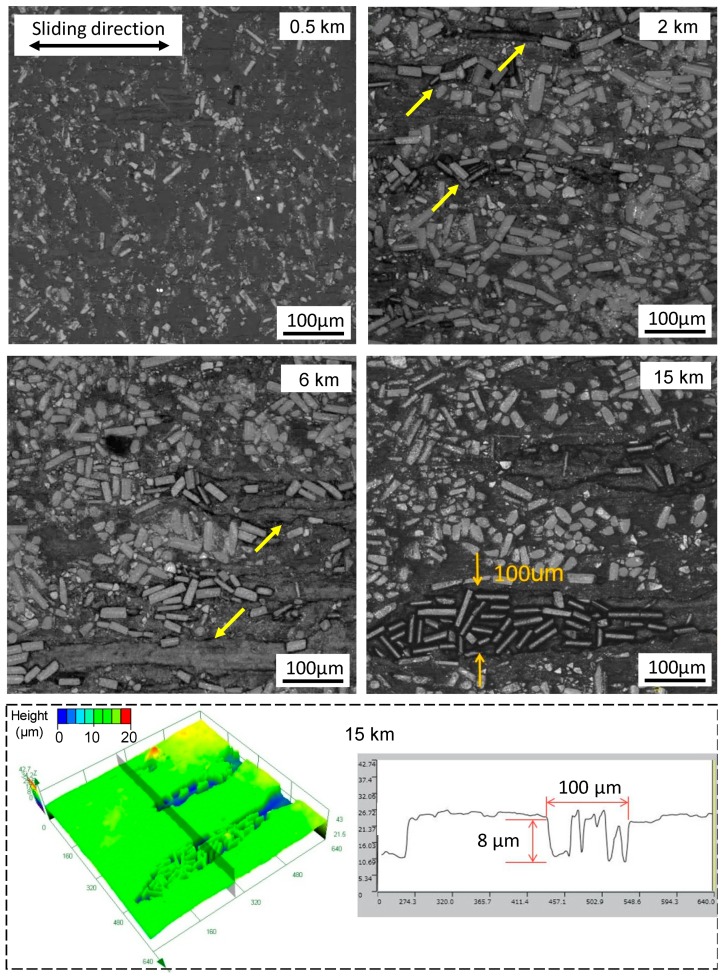
The wear evolution of the GFRPTFE sliding against duplex steel at 200 °C, and the 3D surface profile of the specimen after 15 km sliding.

Figure 8 illustrates the observation on the wear process of the GFRPTFE, which was performed at 200 °C. The initial wear behaviors are still the removal of top layer PTFE and the exposure of glass fibers which have been found at 100 °C. As compared, however, to the surface morphology in the transition region (see the images at 2 km in Figure 7 and Figure 8), two different phenomena arise: (1) The fibers subjected to the reciprocating friction force begin to debond from the matrix, and trend to alignment with the sliding direction, and (2) the micro plough grooves are presented on the worn surface as a result of fiber scratching on PTFE. As can be seen in the 6 km image, the plough grooves propagate longitudinally and laterally with the periodical reciprocating sliding. Eventually the neighboring grooves may join together and form a large-scale ravine, and the peeled-off fibers and wear dusts may be trapped in the ravine as well (see the last CLSM image). Note that the formation of the ravine and the filling process probably occur simultaneously, though no experimental evidence is provided in this study. The 3D surface profile can give a better illustration; the depth of the ravine is about 8 µm which is almost equal to the height of the debonded glass fibers.

### 3.4. The Development of the Transfer Film on the Steel Counter-Face at Elevated Temperature

**Figure 9:**
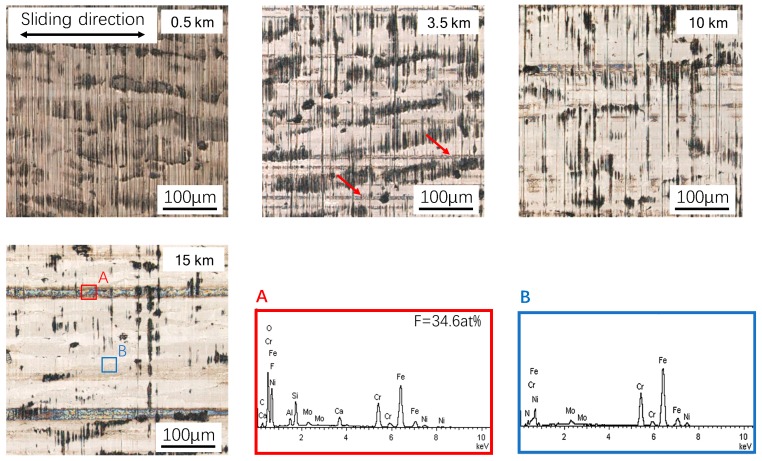
The typical confocal laser scanning microscopy (CLSM) images of the counter-part surface showing the transfer film development at 100 °C, and the chemical composition after 15 km sliding.

The surface morphological changes of the cylinder liner specimen were also observed to better understand the wear behavior of the counter-part. The representative CLSM images of the counter-part surface showing the transfer film development at 100 °C are presented in Figure 9. The first image, on which the vertical honing pattern can be seen, is close to the as-received inner surface of the cylinder liner. The wear dusts can be produced as the sliding distance increases, and can be accumulated in the machining stripes. Meanwhile the streaked transfer films along the sliding direction are generated as marked in the image of 3.5 km in Figure 9. The subsequent behaviors include: (1) The grinding of the counter-part surface, as a result of which the honing patterns gradually disappear; and (2) the growth of the transfer film in both width and length. After 15 km sliding, the surface becomes much smoother and the tribo-film with a width of ~20 µm can be observed. Compared with the polished body material of the cylinder liner specimen (blue box), the chemical composition of the tribo-film is quite different (red box). Besides Fe, the dominating element is F, which is definitely from the GFRPTFE, while the existence of O indicates that oxidation occurs during sliding. As a result, the transfer film due to tribology is formed Note that the cylinder liner specimens were cleaned ultrasonically in acetone before the CLSM observation; no detachment of the film implies its good bonding with the liner specimen. While the morphological evidence clearly proves the presence of the transfer film, the film is not considered to play a significant role during the sliding due to the low area ratio on the surface.

**Figure 10:**
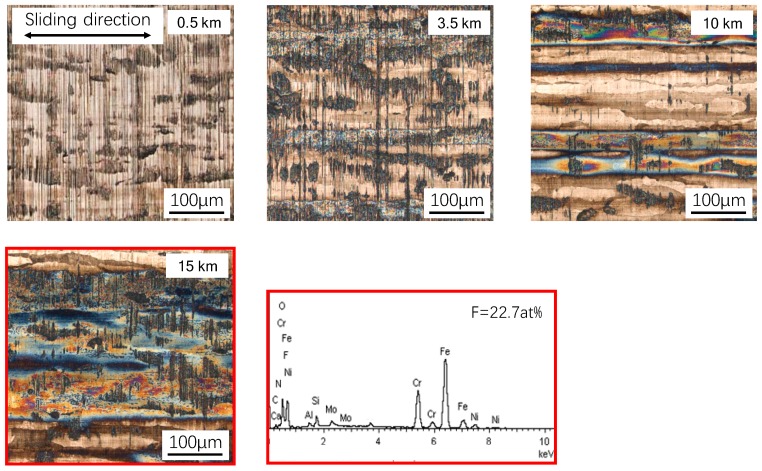
Typical CLSM images of the counter-part surface showing the transfer film development at 200 °C, and the chemical composition after 15 km sliding.

From the observation of images selected at different sliding distances under 200 °C (see Figure 10), it can be found that the wear behaviors are qualitatively similar to those observed at 100 °C. The most noticeable differences are: (1) The area of the transfer film is much larger than that of 100 °C at a given sliding distance; (2) the polishing process of the counter-part surface is faster as well due to the higher friction force; (3) more wear dust can be produced and collected on the surface (compare the images of 0.5 and 3.5 km). As can be seen in the image of 10 km, the area occupied by the transfer film is not much less than that of the metallic surface, and the polished surface that is found after 15 km sliding under 100 °C already exists. Interestingly, there are some island-like substrate materials inside the tribo-film, which are not just due to the wider streak, like the ones formed at 100 °C, and which prevent the abrasive wear of the GFRPTFE [2]. It also can be deduced that the high temperature promotes the formation of the transfer film. However, the transfer film cannot cover the whole surface, even at 200 °C. From the image of 15 km, the smooth steel surface can still be observed, and the majority element is still Fe. So the formed tribo-film may also be abraded and destroyed by the sharp and hard glass fibers during the sliding [2].

## 4. Discussion

In this section, wear mechanisms of the GFRPTFE sliding against steel at temperatures lower and higher than Tg, and their correlation with the friction coefficient and wear rate will be discussed. 

**Figure 11:**
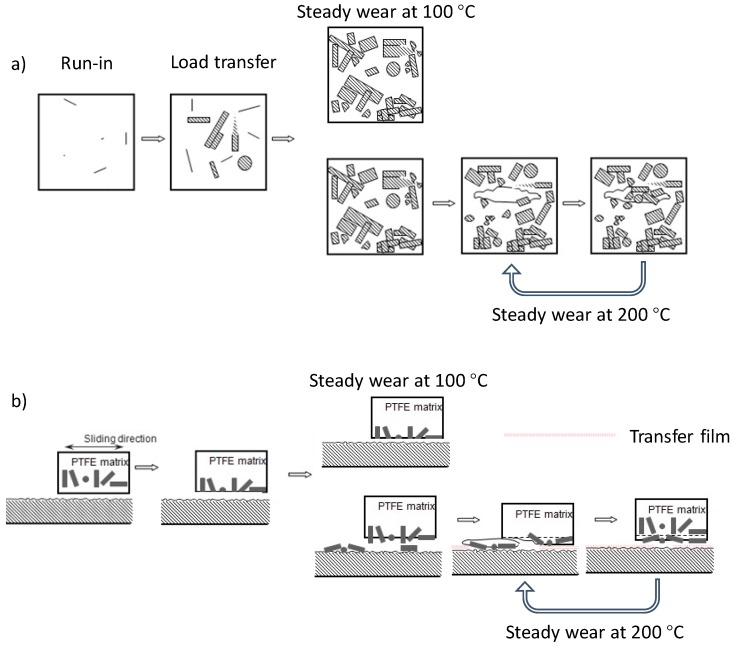
The schematic of the wear evolution of GFRPTFE sliding against steel counter-face. (**a**) Top view and (**b**) cross-section view.

The initial run-in period, where the wear rate is comparatively high, is characterized by the micro-plowing and micro-cutting of the PTFE on the surface of the ring specimen. At the beginning of the sliding, the glass fibers are almost fully buried beneath the top PTFE layer, so only the PTFE is in direct contact with the steel counter-face (see Figure 11, run-in period). As a result, the asperities on the counter-face can easily cut the PTFE, causing the relatively high wear rate. In addition, the nature of viscoelasticity induces the higher viscosity, but lowers the young’s modulus and the yield strength as well as the shear strength of PTFE under higher temperatures, so the friction coefficient and the wear rate are increased with the temperature (see Figure 3 and Figure 4). 

After the removal of the top PTFE layer, the embedded glass fibers begin to be exposed. Then, with the increase of the area ratio glass fiber on the GFRPTFE surface, the load is transferred to the glass fiber from the PTFE. As the glass fibers can enhance the overall shear strength of the surface (based on the rule of mixtures), they reduce the subsurface deformation and obstruct the propagation of cracks [3]; the wear rate decrease and the glass fiber becomes the main load-bearing constituent. Regarding the friction coefficient, the exposure of glass fiber enhances the strength of the GFRPTFE surface, but the higher temperature softens the PTFE matrix as well [20]. As a result, the plastic flow of the PTFE matrix that is observed in the image of 2 km of Figure 8 greatly increases the friction force. In contrast, the glass fibers are strongly bonded with the matrix under temperatures lower than Tg (see Figure 5), because the rigid amorphous segments are restricted, and the PTFE matrix is very likely deformed elastically. Therefore, the friction coefficient is low.

With the increase of the sliding distance, the wear rate trends to stability. No more phenomena were found for the low temperature, and both the friction coefficient and wear rate are low. However, 200 °C is much higher than Tg, thus the amorphous segments are prone to move, and the high temperature loosens the restraint of crystal lattice on the polymer chains. Thus, the macroscopic properties of PTFE matrix, such as modulus and strength, decrease dramatically, resulting in the detachment of the glass fibers and very complicated wear behaviors. Besides the continuous exposure of the inner layer fibers, worn fibers are peeled off when the bond between the fillers and the PTFE matrix is loosened, which brings two disadvantages: The abrasion of the GFRPTFE and the starching of transfer film on the counter-face; ineffective reinforcement of fibers in PTFE decreasing the wear properties of the PTFE composites was also reported [7]. This is because the debonding fibers can easily serve as the third body on the interface between the friction pair. On the other hand, there are a number of air voids inside the GFRPTFE specimen (see Figure 2), and they come out with the increase in wear depth. These voids are the ideal source of cracks, they may initiate cracks and propagate damages like large-scale flakes and grooves. Interestingly, the third body fibers also tend to be trapped in the flake delamination as discussed in Section 3.3. Moreover, reformation of the transfer film may occur as well [2]. This is because the tribo films change with sliding distance; the ratio, however, can almost keep constant. Therefore, it can be concluded that the steady wear period under high temperature is dynamically stable and that dominant behaviors may change with the sliding distance, resulting in the slight fluctuation of wear rate and the large deviation of the measured wear rate after 15 km sliding. However, the glass fibers bear the majority load regardless of the micro behaviors and temperature, hence the low wear rate and steady-state conditions can be maintained.

From the findings of this study, it can be found that softening of the PTFE matrix and detachment of the glass fibers are the main reasons that worsen the tribological properties of GFRPTFE at elevated temperatures. Aiming at these problems, using the cross-linked PTFE possessing the enhanced high temperature performance and the better coupling agent for the matrix and glass fiber interface may be the feasible solution.

## 5. Conclusions

The main conclusions were drawn as follows:

The coefficient of friction initially increases with the sliding distance and then falls into the stable stage. Based on the wear behaviors, three regions, run-in, transition, and steady wear, can be identified regardless of the temperature. The relationship between the wear rate and the sliding distance shows the inverse trend compared with the friction coefficient. But in the steady wear region, both the friction coefficient and the wear rate increase with the increase of temperature.

From the DSC measurement, the Tg of the GFRPTFE in this study is ~121 °C. thus the best working condition for the composite is lower than Tg with respect to the tribological performance. Regarding to the formation of tribo-film, high temperature is a beneficial factor.

The wear evolution can be summarized as: The direct contact of PTFE and steel in the run-in stage causes the high wear rate. After the removal of the top PTFE layer, the buried glass fibers begin to be exposed and gradually become the main load-bearing constituent. As a result, the wear rate decreases sharply. With the further increase of the sliding distance, the wear rate trends to stability for lower temperatures. The debonding of the fibers, however, occurs due to the softening of the PTFE matrix, bringing the new wear behaviors, including: (1) The third body abrasion, (2) flake delamination of the PTFE matrix, (3) scratching and reformation of transfer film on the counter-face, and (4) the filling of the large scale PTFE groove. These behaviors dominate the different stages in the stable region, but occur simultaneously and cause the dynamic steady wear. As a result, the wear rate at 200 °C is slightly fluctuant.

## Figures and Tables

**Table 1:** The engineering information and physical data of the glass fiber-reinforced Polytetrafluoroethylene (GFRPTFE) specimens.

Engineering Information	
Temperature-Typical Range (°C)	−240/+288
Maximum PV (continuous) (MPa·m/s)	10,000 (0.35) *
Maximum P-psi (static) (MPa)	1000 (6.9) *
Maximum V-SFM (no load) (m/s)	400 (2) *
Water Absorption ASTM D570	0%
Flammability ASTM D635	Non-Flammable
Chemical Resistance	Inert
Thermal Conductivity (W/m·k)	0.33
Thermal Expansion (26 °C to 149 °C) (×10^−5^ m/m °C)	Length 11.1
Elongation ASTM D4894	175%
Tensile Strength ASTM D4894 (MPa)	2000 psi (13.8) *
Deformation ASTM D621	5% (1500 psi-24 h·RT)
Specific Gravity ASTM D792	2.22

*: metric data in parentheses.

**Table 2:** The dimensions of the GFRPTFE specimens and steel cylinder liner specimens.

Tested Specimens	Axial Length (mm)	Inner Diameter (mm)	Outer Diameter (mm)
GFRPTFE	2	78	90
Cylinder liner	63	90	100

## References

[1] Khare H.S., Moore A.C., Haidar D.R., Gong L., Ye J., Rabolt J.F., Burris D.L. (2015). Interrelated Effects of Temperature and Environment on Wear and Tribochemistry of an Ultralow Wear PTFE Composite. J. Phys. Chem. C.

[2] Song F., Wang Q., Wang T. (2016). Effects of glass fiber and molybdenum disulfide on tribological behaviors and PV limit of chopped carbon fiber reinforced Polytetrafluoroethylene composites. Tribol. Int..

[3] Blanchet T.A., Kennedy F.E. (1992). Sliding wear mechanism of polytetrafluoroethylene (PTFE) and PTFE composites. Wear.

[4] Kim S.S., Shin M.W., Jang H. (2012). Tribological properties of short glass fiber reinforced polyamide 12 sliding on medium carbon steel. Wear.

[5] Conte M., Igartua A. (2012). Study of PTFE composites tribological behavior. Wear.

[6] Cheng X.-H., Xue Y.-J., Xie C.-Y. (2002). Friction and wear of rare-earth modified glass-fiber filled PTFE composites in dry reciprocating sliding motion with impact loads. Wear.

[7] Shi Y., Feng X., Wang H., Lu X. (2007). Tribological properties of PTFE composites filled with surface-treated carbon fiber. J. Mater. Sci..

[8] Mu L., Xin F., Zhu J., Wang H., Sun Q., Shi Y., Lu X. (2010). Comparative Study of Tribological Properties with Different Fibers Reinforced PTFE/PEEK Composites at Elevated Temperatures. Tribol. T..

[9] Yuan Q., Jun G., Wenhan C., Honggang W., Junfang R., Gui G. (2018). Tribological Behavior of PTFE Composites Filled with PEEK and Nano-Al_2_O_3_. Tribol. T..

[10] Makowiec M.E., Blanchet T.A. (2017). Improved wear resistance of nanotube- and other carbon-filled PTFE composites. Wear.

[11] Qiu M., Yang Z., Lu J., Li Y., Zhou D. (2017). Influence of step load on tribological properties of self-lubricating radial spherical plain bearings with PTFE fabric liner. Tribol. Int..

[12] Sripakagorn A., Srikam C. (2011). Design and performance of a moderate temperature difference Stirling engine. Renew. Energy.

[13] Shen Y., Yu B., Lv Y., Li B. (2017). Comparison of Heavy-Duty Scuffing Behavior between Chromium-Based Ceramic Composite and Nickel-Chromium-Molybdenum-Coated Ring Sliding against Cast Iron Liner under Starvation. Materials.

[14] Ma S., Chen W., Li C., Jin M., Huang R., Xu J. (2018). Wear Properties and Scuffing Resistance of the Cr–Al_2_O_3_ Coated Piston Rings: The Effect of Convexity Position on Barrel Surface. J. Tribol..

[15] Shen Y., Lv Y., Li B., Huang R., Yu B., Wang W., Li C., Xu J. (2019). Reciprocating electrolyte jet with prefabricated-mask machining micro-dimple arrays on cast iron cylinder liner. J. Mater. Process. Technol..

[16] Kato H., Komai K. (2007). Tribofilm formation and mild wear by tribo-sintering of nanometer-sized oxide particles on rubbing steel surfaces. Wear.

[17] Harris K.L., Pitenis A.A., Sawyer W.G., Krick B.A., Blackman G.S., Kasprzak D.J., Junk C.P. (2015). PTFE Tribology and the Role of Mechanochemistry in the Development of Protective Surface Films. Macromolecules.

[18] Calleja G., Jourdan A., Ameduri B., Habas J.P. (2013). Where is the glass transition temperature of poly(tetrafluoroethylene)? A new approach by dynamic rheometry and mechanical tests. Eur. Polym. J..

[19] Lehnert R.J., Hendra P.J., Everall N., Clayden N.J. (1997). Comparative quantitative study on the crystallinity of poly(tetrafluoroethylene) including Raman, infra-red and 19 F nuclear magnetic resonance spectroscopy. Polymer.

[20] Song F., Yang Z., Zhao G., Wang Q., Zhang X., Wang T. (2017). Tribological performance of filled PTFE-based friction material for ultrasonic motor under different temperature and vacuum degrees. J. Appl. Polym. Sci..

